# Multifunctionality Analysis of Structural Supercapacitors— A Review

**DOI:** 10.3390/ma17030739

**Published:** 2024-02-03

**Authors:** Willi Zschiebsch, Yannick Sturm, Michael Kucher, Davood Peyrow Hedayati, Thomas Behnisch, Niels Modler, Robert Böhm

**Affiliations:** 1 Faculty of Engineering, Leipzig University of Applied Sciences, PF 30 11 66, 04251 Leipzig, Germany; yannick.sturm@stud.htwk-leipzig.de (Y.S.); michael.kucher@htwk-leipzig.de (M.K.); davood.peyrow_hedayati@htwk-leipzig.de (D.P.H.); robert.boehm@htwk-leipzig.de (R.B.); 2 Institute of Lightweight Engineering and Polymer Technology (ILK), Technische Universität Dresden, Holbeinstraße 3, 01307 Dresden, Germany; niels.modler@tu-dresden.de

**Keywords:** structural supercapacitors, multifunctional energy storage composite (MESC), carbon fiber electrode, structural electrolyte, separator

## Abstract

Structural supercapacitors (SSCs) are multifunctional energy storage composites (MESCs) that combine the mechanical properties of fiber-reinforced polymers and the electrochemical performance of supercapacitors to reduce the overall mass in lightweight applications with electrical energy consumption. These novel MESCs have huge potentials, and their properties have improved dramatically since their introduction in the early 2000’s. However, the current properties of SSCs are not sufficient for complete energy supply of electrically driven devices. To overcome this drawback, the aim of the current study is to identify key areas for enhancement of the multifunctional performance of SSCs. Critical modification paths for the SSC constituents are systematically analyzed. Special focus is given to the improvement of carbon fiber-based electrodes, the selection of structural electrolytes and the implementation of separators for the development of more efficient SSCs. Finally, current SSCs are compared in terms of their multifunctionality including material combinations and modifications.

## 1. Introduction

The ongoing global warming, the scarcity of resources, and the environmental crisis are changing the way humanity thinks about energy, in particular energy for transportation. Considering the electrification of transportation vehicles as one promising approach for the decarbonization of the transport sector, the demand for more efficient energy storage rises [1]. With motors achieving close to maximum efficiency and electrical storage systems nearing the thermodynamic limit of energy density [2], a major factor that can still be improved is the weight of vehicles, where a reduction results in a lower overall energy consumption [3].

There are two main approaches for structural electrical energy storage (EES) systems. A classical approach is to increase the specific energy density of the active material in order to store the same amount of energy within a smaller portion of EES [4]. A novel approach is to integrate EES into lightweight structures such as structural components made of carbon fiber-reinforced polymers (CFRPs), which can be used for energy storage and load bearing at the same time. The results of this approach are called multifunctional energy storage composites (MESCs). The MESC concept results in the reduction in the monofunctional weight-bearing elements of the EES and therefore increases the overall specific energy density [5].

Currently, supercapacitors (SCs) and conventional lithium-ion batteries (LiBs) are the two main types of EES. While LiBs are based on Faradaic reactions, SCs present a compromise between classical capacitors and batteries by storing charges electrostatically in an electric double layer and can also employ other mechanism, such as Faradaic processes when transition metal oxides are available on the electrode surface [6]. The electrochemical performance is often characterized by specific capacitance, energy density, and power density [6]. Additionally, mechanical properties that are important for multifunctionality include the Young’s modulus and the tensile strength. Furthermore, CFRPs are frequently characterized by more intricate failure criteria such as delamination or matrix cracking [7]. To evaluate the SSCs’ electrochemical performance in addition to energy density and power density, other material parameters such as ionic conductivity or specific surface area (SSA) are taken into consideration. In general, SCs are attractive candidates for use in structural EES systems due to their simple design, high power density, and long cycle life [8].

**Figure 1:**
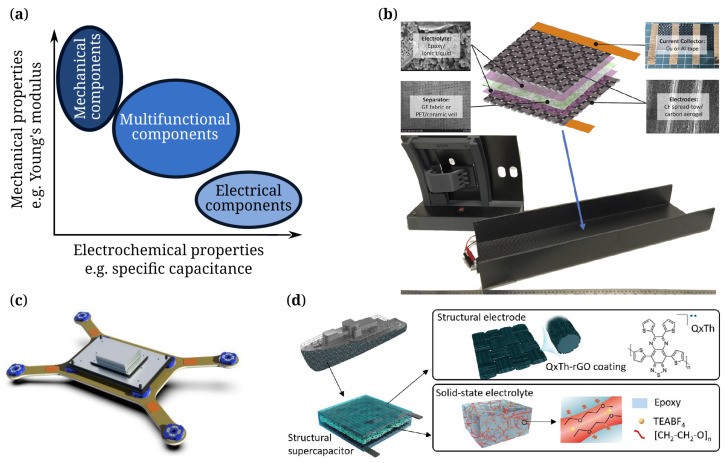
(**a**) Schematic of multifunctionality as a combination of high mechanical and high electrochemical properties, (**b**) structural supercapacitor constituents inside a multifunctional fuselage beam demonstrator [12], (**c**) multifunctional highly integrated satellite panel powered by structural supercapacitors [13], (**d**) boat hull with integrated structural supercapacitors [14].

As mentioned above, SSCs are a promising storage technology for the electrification of the transport sector and spacecraft, especially when fast charging and discharging cycles need to be realized and minimal weights are required. The first carbon fiber (CF)-based structural capacitor was built in 2001 [9]; however, the term “SSCs” was first introduced by the United States Army in 2008 [10], but similar ideas on multifunctional materials have been circulated since at least 2003, as investigated by Christodoulou and Venables [11]. Currently, initial concept studies have been demonstrated for fuselage beams [12], satellite panels [13], and composite boat hulls [14] (Figure 1b–d). One of the first studies on the mechanical and electrical behavior of CF structural capacitors including the effects of delamination and interlaminar damage was conducted by Shen and Hongyu in 2017 [15] and prediction of the mechanical behavior of SSCs was carried out by Valkova et al. in 2022 [16].

**Figure 2:**
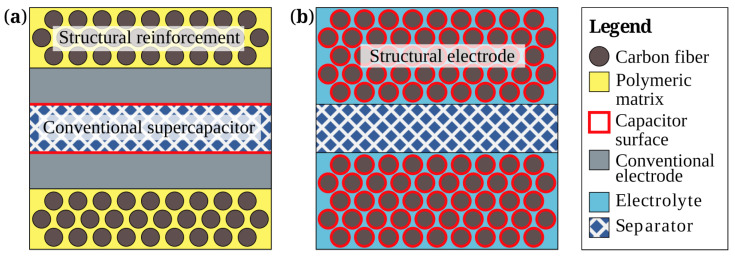
Different design concepts of currently applied SSCs: (**a**) integrated supercapacitor, (**b**) laminated structural supercapacitor.

For SSCs, there are the following design concepts:

(a) Integrated SSC: In the integrated configuration, SSCs are developed by sandwiching monofunctional SCs between two composite layers of structural reinforcement laminates (Figure 2a) [17]. SSCs could be created by packing monofunctional SCs inside structural reinforcement layers made of CFs [8], glass fibers (GFs) [18], or aramid fibers [19]. The most common design is composed of CF electrodes separated by a separator layer connected by an ion-conducting electrolyte [20]. While such a device displays electrical storage and load-bearing functionality, true multifunctionality is not given, since the respective parts of the SSC do not provide multifunctional properties. In addition to CFRPs, other types of reinforcement have been reported in the literature, such as the integration of SCs into a honeycomb structure with the aim to further improve the lightweight properties [21].
(b) Laminated SSC: An SSC with a higher integration level includes the use of CF mats acting simultaneously as electrode and reinforcement. For this certain design, a polymer-based ionic liquid functions as both composite matrix and the electrolyte material (Figure 2b).

The current study provides an overview of different approaches to improve SSCs’ electrochemical and mechanical performance with regards to their overall multifunctionality with the goal to decrease the structure’s total weight. By analyzing different parts of the SSC CF electrode, structural electrolyte, and separator and examining the respective influences on their multifunctionality performance, potential areas of improvement are found. Thereby, the novelty of the current study is the investigation of both the mechanical and electrochemical properties in terms of multifunctionality. Furthermore, an overview of the materials and methods to further improve the performance of SSCs is provided. In the following sections, the individual components of SSCs are reviewed using selected research results and applicable improvement areas are introduced.

## 2. Materials and Methods

### 2.1. Data Sources and Selection

Data were collected from publications in reputable scientific databases including MDPI, IEEE Xplore, ScienceDirect, and Web of Science. These databases were systematically screened using the keywords “structural”, “supercapacitors”, “electrode materials”, “electrolytes”, “separator”, and “energy storage”, ensuring the inclusion of studies addressing the multiple aspects of structural supercapacitor design and functionality. Emphasis was placed on selecting studies that presented novel methodologies, experimental validations, and theoretical frameworks, thereby enriching the literature review with a robust and diverse set of perspectives. The selection criteria prioritized studies that delved into the synergistic effects arising from the combination of different materials and investigations exploring the impact of various fabrication techniques on supercapacitor performance. Special attention was given to studies that provided a comprehensive analysis of the electrochemical and structural properties of SSC components.

### 2.2. Assessment of Multifunctionality

According to Ashby’s multi-objective optimization approach [22], the unitless multifunctional efficiency of an SSC $\eta_{\mathrm{SSC}}$ depends on the relative electrical efficiency $\eta_{E}$ and relative structural efficiency $\eta_{M}$ [23]. To evaluate the multifunctionality, the criteria proposed by Zhou et al. [17] was used. This criteria is defined as the ratio between the current properties $x_{i}$ and a chosen benchmark value $X_{i}$. To calculate the multifunctional efficiency of SSCs, a summation of the ratios of the mechanical and electrical properties is used:

(1) $$\eta_{\mathrm{SSC}}=\eta_{M}+\eta_{E}=\frac{x_{M}}{X_{M}}+\frac{x_{E}}{X_{E}}.$$

Hereby, $x_{M}$ and $x_{E}$ correspond to the mechanical and electrochemical properties of the SSC, while $X_{M}$ and $X_{E}$ are benchmark values for the mechanical and electrochemical performance. Benchmark values recommended by Snyder et al. [24] were applied, which represent realistic values for SSCs. In the current study, the benchmark values for structural properties are based on the properties of a satin weave CF fabric (T300 3k 8-harness, Fibre Glast Developments Corporation, Brookville, OH, USA) in an epoxy matrix [25], which has a Young’s modulus value of $E_{\left|\right|}$ = 42.7 GPa for the parallel unilateral (UD) lamina, a shear modulus parallel–transverse of $G_{||⊥}$ = 2.93 GPa, a tension strength of $R_{\left|\right|}^{t}$ 800 MPa for the parallel UD lamina, and a flexural strength $R_{||⊥}$ of 850 MPa [25]. Additionally, the electrochemical benchmark value is based on commercial datasheets of a commercial SC (BCAP3000, Maxwell Technologies, San Diego, CA, USA) with an energy density of 6000 mWh/kg [24].

By using the proposed method, the highest efficiency for either mechanical or electrochemical properties is $\eta_{M}$ = $\eta_{E}$ = 1, resulting in an overall efficiency of $\eta_{\mathrm{SSC}}$ = 2. However, all values of $\eta_{\mathrm{SSC}}$ > 1 represent additional multifunctional efficiency, which results in mass savings. SSCs with a multifunctional efficiency below 1 could theoretically be replaced by a lighter structure using the benchmark SC and the satin weave CF fabric composite mentioned above for structural support.

## 3. Component Analysis

### 3.1. Electrode Material

The electrodes and its interface are responsible for the amount of charge stored inside the SC. Depending on the type of SC, the electrode can either electrostatically store charges for electric double-layer capacitors (EDLCs), undergo charge transfer reactions in pseudocapacitors, or perform both functions in hybrid capacitors. Porous carbon-based materials are commonly employed as electrode materials in EDLCs [26]. The capacitance of EDLC devices primarily originates from the accumulated charges at the interface between the electrode pores and the electrolyte [27,28]. Therefore, parameters such as high SSA, high electrical conductivity, high redox activity, and cycling behavior are crucial properties for SC performance. Typical electrode materials with pseudocapacitive behavior are often associated with transition metal oxides (MOx), such as ${\mathrm{RuO}}_{2}$ [29,30] and ${\mathrm{MnO}}_{2}$ [31,32], in aqueous electrolytes [26]. Although transition metal oxides frequently do not possess the mechanical properties suitable for direct usage in SSCs, they are commonly combined with carbon-based materials, such as carbon nanotube nickel (CNT-Ni) foams [33,34], to enhance their performance.

**Figure 3:**
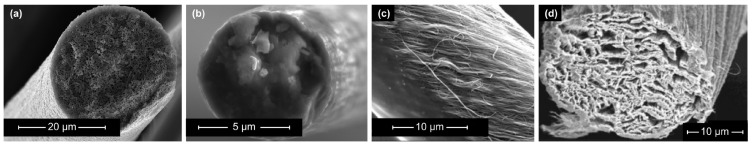
Scanning electron microscopy images of the microstructure of selected carbon-based electrode materials: (**a**) porous carbon fiber manufactured by the “Research Center Carbon Fibers Saxony” (RCCF) of TU Dresden, (**b**) single fiber of carbon roving (Tenax HTA 5131 800tex, Teijin Carbon Europe, Wuppertal, Germany) (**c**) carbon nanotube fibers (reproduced from [35]), (**d**) graphene fiber (reproduced from [36]).

Promising material candidates for usage as electrode materials in SSCs include CFs (Figure 3b), CNT fibers (Figure 3c), and graphene fibers (Figure 3d). These materials exhibit extraordinarily high strength, high Young’s modulus values, high SSA, and high electrical conductivity. However, large-scale fabrication methods to produce CNT or graphene fibers are currently challenging and need to be further developed.

In comparison with conventional CFs, the Young’s modulus of graphene fibers is lower [37,38]. Nevertheless, the first implementation of graphene fibers into an all-solid-state SSC was performed by Senokos et al., which resulted in high multifunctionality [8]. In general, the application of CNT fibers and graphene fibers as electrode material in SSCs can potentially obtain comparably high mechanical and electrochemical performances [39,40,41]. Additionally, a promising feature is that guest materials can directly be embedded into these fibers, which leads to an increase in the electrochemical performance [42].

As mentioned above, carbon-based materials are widely utilized as the primary material for the electrodes in SCs owing to their favorable polarizability and ability to withstand high temperatures. It appears that the most practical choice for SSC electrodes are carbon-based fibers. There are various SSC electrode fabrication techniques, which involve the utilization of nonwoven CF mats [43], UD CFs [44], or woven CF mats [45] (Table 1). Although nonwoven CF mats exhibit lower mechanical strength compared to the other two, they can still be considered to be suitable for implementation in SSCs due to their higher SSA. This leads to enhanced power and energy densities. Woven CF fabrics and UD CFs, on the other hand, can withstand much higher forces, whereby UD fibers can bear high loads in one certain direction. Additionally, they both can conduct electricity, and thus they can act as current collectors [42].

**Figure 4:**
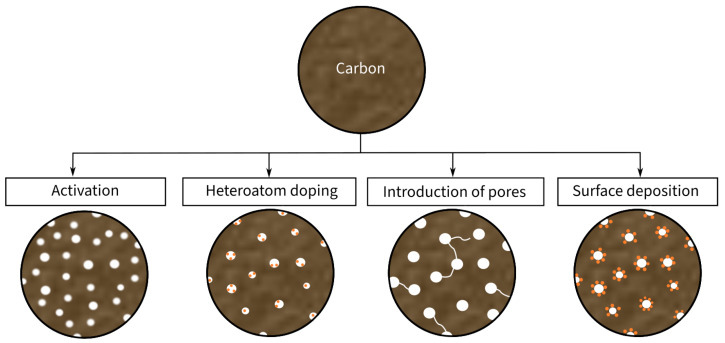
Schematic illustration of the modifications of carbon material for the usage as electrode material.

Conventional CFs (Figure 3b) are characterized by a relatively dense structure of multiple graphite layers, which results in high mechanical properties and relatively good electrical conductivity. However, they provide low electrochemical properties in comparison to other carbon-based fibers [46]. As an alternative solution, porous CFs (Figure 3a) have been recently introduced as a candidate electrode material in SSCs. Porous CFs have a higher SSA due to an intentional increase in the material’s porosity. As a result of this increase, the specific capacitance rises at the expense of a decline in mechanical properties. Consequently, the ongoing research focuses on improving the SSA and electrical conductivity of CF-based electrodes while retaining high mechanical properties. According to Frackowiak and Béguin [47], there are different methods, such as activation, heteroatom doping, and surface deposition, for enhancing the SSA of carbon materials, which are summarized in Figure 4. These modifications are explained in more detail in the following sections.

#### 3.1.1. Increasing Carbon Fiber Porosity

In addition to the activation of CFs after fiber fabrication and the method of heteroatom doping, there are two other techniques to introduce pores into CFs during fiber fabrication. The two techniques are carbonization via activation of the precursor fibers and the introduction of pores in precursor fibers via pore-forming agents [42,48]. Carbonization describes the heating process of the precursor fiber inside a reactive gas, where the gas reacts with the carbon and forms a porous structure. This technique can increase the power density of SCs by 3–4 times [49].

Another method to introduce pores into CFs during the fabrication process is via the use of solid organic or inorganic pore-forming agents. Organic pore-forming agents such as polymethyl methacrylate (PMMA), polyvinylpyrrolidone (PVP), or sulfonated tetrafluoroethylene based fluoropolymer-copolymer can be mixed or electrospun into the precursor material and are later burned out during the carbonization process [42]. Due to the decomposition of the organic pore-forming agents, pores form inside the CFs [50]. For inorganic agents like acids, alkalis, and salts, different methods such as etching or bubbling of formed gases lead to the creation of a porous structure [42]. As mentioned above, the resulting porous CF electrodes have better electrochemical properties, but inferior mechanical properties. Nevertheless, a study by Peng et al. suggests that the porous CF can indeed contribute in a positive way towards the overall mechanical properties of SSCs by increasing the interlaminar shear strength between the CFs and the polymer by 70% [51]. Therefore, efforts in the stud of fabrication methods are mainly focused on developing highly flexible porous CFs [52].

#### 3.1.2. Activation of Carbon Fibers after Fabrication

An activation step after CF production can increase the SSA. Several key methods of CF activation are summarized in the following. The CF activation processes include thermo-chemical, wet chemical, electrochemical, and physical activation. All these activation processes create CFs with a high SSA. In addition, they result in the creation of functional groups on the CF surface to enhance pseudocapacitance [42].

Thermo-chemical activation is the thermal treatment of CFs under the presence of different chemicals. The result depends on the chemical type, amount, and exposure time to CFs during the activation process. Since this process increases the porosity, the mechanical properties such as tensile strength usually diminish [42]. Nevertheless, there is a potential use for thermo-chemical-activated CFs as electrodes in structural energy devices, where results showed a 100-fold increase in specific surface area and a 50-fold improvement in specific electrochemical capacitance without any degradation of the fibers’ mechanical properties [53].

Wet chemical activation does not require thermal activation and instead relies on more aggressive chemicals such as oxidants, acids, or alkalis. Results of the CF activation with $H_{2}{\mathrm{SO}}_{4}$ showed a 15 times increase in SSA [54]. In addition to a higher SSA, this kind of activation process can lead to a better interfacial adhesion between fibers and matrix material [55]. In a study by Chen et al., the post-activated surface microstructure of the CF electrodes allowed for a better penetration of the electrolyte and thus resulted in better ion diffusion [56]. However, this was achieved at the cost of deteriorated electrical conductivity and mechanical strength of treated CFs [42].

Electrochemical activation, such as electrochemical oxidation, presents a simple, environmentally friendly and fast way to increase the specific capacitance of CF-based energy devices [42]. This is due to the establishment of a hierarchic porous structure with micropores for charge storage and mesopores for the creation of high-speed pathways for ion transfer to the inner surface of the CF electrodes. Furthermore, the creation of functional groups generates pseudocapacitance and improves the electrical conductivity [57,58].

Unlike other activation methods, a study by Liu et al. showed an increase in the tensile strength of the treated CFs and better interfacial adhesion between CFs and the polymeric matrix by 16.6% and 8.6% [59], respectively.

The physical activation of CFs is characterized by irradiation, such as plasma, gamma rays, ultraviolet (UV) light, or electron beams, in various atmospheres to stimulate chemical reactions [60]. These reactions lead to the formation of a higher SSA and the creation of functional groups [42]. A study by Okajima et al. on treated CFs showed an improvement in the capacitance of 28%, which was attributed to more functional groups and an increase in SSA of 34% [61]. Additionally, a study by Xiao et al. has shown that physical activation via gamma irradiation can improve the mechanical properties of CFs due to cross-linking effects between graphene layers in the CFs [62].

#### 3.1.3. Heteroatom Doping of Carbon Fibers

Heteroatom doping with nitrogen, oxygen, boron, phosphorus, and sulfur or co-doping with a mixture of heteroatoms leads to the deformation of a graphitic network caused by the atomic size difference between the incorporated atoms and the host carbon atoms [63]. This induced deformation results in changes to SSA, pore size distribution, and pore volume, which can have a positive effect on the electrochemical performance. Other effects that positively influence the properties of the electrodes are the creation of a hydrophilic surface due to nitrogen functional groups, the establishment of a positive charge density due to the higher electronegativity of the heteroatoms, and a substantial increase in active sites caused by the induction of local defects [64,65]. Additionally, elemental doping with heteroatoms can significantly increase the pseudocapacitance of SCs, leading to a better overall capacitance of the energy storing device [57,66]. Additionally, co-doping can further increase the SSA through synergies of different heteroatoms, with an almost two-fold increase in the SSA and pore volume [64].

Similar to other CF treatments, the introduced porosity via doping methods will negatively influence the CFs’ mechanical properties. Therefore, the production of heteroatom-doped CFs with both high pore volume and tensile strength and stiffness is a challenging task [67].

#### 3.1.4. Surface Deposition of Carbon Fibers (Hybrid Electrodes)

One of the most common methods of improving the electrochemical properties of SC electrodes is the deposition of active materials onto the base electrode material to create a so-called “hybrid electrode”. In SSCs, the CF electrodes can serve as an excellent carrier material for active materials, which can enhance the electrochemical properties. Typical guest materials for CFs are carbon-based nanomaterials such as CNTs and graphene, conducting polymers (CPs), metal oxides, carbon aerogels (CAGs), or a mixture of different active materials [42].

CNTs have been widely used as an electrode material for EES systems due to their excellent specific surface area of up to 1600 $m^{2}/g$, high electric conductivity, and a wide potential range [68,69,70]. Additionally, CNTs have outstanding mechanical properties such as a high Young’s modulus and high strength [71]. Therefore, several studies have shown a significant increase in specific capacitance when CFs were either coated with CNTs or CNTs were directly grafted onto carbon electrodes [72]. However, due to surface damage of the fibers during the CNT grafting process, the overall mechanical strength can be significantly reduced, while the Young’s modulus stays almost unaffected [73]. Nevertheless, Zakaria et al. were able to improve the tensile strength and Young’s modulus of CFs by depositing dense CNT networks on the fiber surface. Their results show a stronger interfacial adhesion between the fibers and the matrix material [74].

Graphene is a particularly suitable active enhancement material for SC electrodes as well. This is due to the high SSA, outstanding electrical conductivity, and superior mechanical properties of graphene [75]. By interleaving graphene nanoplatelets (GNP) between the CF-based electrodes and the polymer electrolyte, Javaid et al. were able to observe a significant increase in specific capacitance up to values of 8.9–118.7 mF·cm^−3^, energy density up to value of 19.7–263.8 Wh·m^−3^ and normalized in-plane shear modulus with values of 1.7–3.1 GPa [76]. Therefore, graphene, similar to CNTs, can not only improve the electrochemical but also the mechanical properties of SSCs.

Moreover, conducting polymers (CPs) such as Polyaniline (PANI), Polypyrrole (PPy), and particularly poly(3,4-ethylenedioxythiophene)(PEDOT) can potentially increase the pseudocapacitance [77]. CPs can be used as individual SC electrodes but they show poor stability during cycling due to structural swelling and mechanical brittleness. As a solution, CPs can be electrochemically synthesized and simultaneously deposited on CF electrodes in SSCs, which could result in improved charge transfer [78].

Comparatively, the deposition of metal oxides (MO_x_) such as MnO_2_, NiO, and V_2_O_5_ on CF-based electrodes results in a high specific capacitance and high metal-like conductivity which nominates them as another candidate for hybrid electrode material in SSC electrode design [79]. In addition to improved pseudocapacitance, the MO_x_ deposition layer is filled with pores which further improve the SSA and increases specific capacitance [80]. Furthermore, improvements in the electrical conductivity and surface area of carbon-based materials are also beneficial for the charge storage and delivery process [81], resulting in an increased cycle life and a high-rate performance [82]. The higher rate performance though deposition of MO_x_ can amplify pseudocapacitance even more, with a reported surface capacity increase of five times [83]. Recently, a study by Deka et al. has shown a significant improvement in mechanical strength and Young’s modulus of the SSC device due to better interfacial interaction between the CFs and the surrounding matrix material [84].

Another important material in efficient SSC hybrid electrodes are CAGs, which are a three-dimensional network of nanocarbon particles. CAGs provide excellent electrochemical properties such as high SSA and high electric conductivity. Because of their highly porous structure, CAGs are apparently not a suitable material for a standalone electrode in SSCs due to low mechanical properties [85]. However, CAGs can still be used in hybrid form to enhance the electrochemical properties of CF electrodes. In this case, they are either infused into the CFs or used as an impregnation. Qian et al. showed that the incorporation of CAG into the CF electrodes can increase the electrochemical performance by approximately 100-fold [86]. Regarding the effect of CAGs on the mechanical properties of hybrid electrodes, often contradictory results are reported in the literature. Qian et al. suggest that the stiff CAG networks effectively improve the matrix properties and, therefore, increase both the in-plane shear strength and shear modulus [86]. A study by Pernice et al. determined that CAGs cause a brittle fracture and thus decrease the overall mechanical performance of SSCs [20,86].

In conclusion, a combination of two or more of the mentioned methods can be a promising approach to obtain higher electrochemical and mechanical performances. As an example, Hudak et al. reported a high-performing SSC hybrid electrode which combined CNTs with the conducting polymer PANI and obtained a specific capacitance with values between 0.84 $F\cdot g^{-1}$ and 26 $F\cdot g^{-1}$) [78]. Other examples are the combination of CNTs with MO_x_ [68] and the combination of GNPs with CAGs [87].

#### 3.1.5. Summary of Electrode Modification Methods

**Figure 5:**
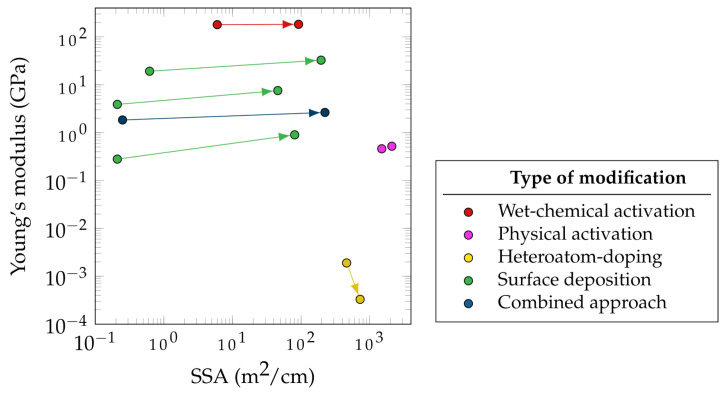
Changes due to different CF-based electrode modifications based on Table A1 [54,61,74,76,84,86,87,88,89,90,91,92].

Figure 5 summarizes the effect of different electrode modification approaches on the electrochemical and mechanical performances of a selected group of electrode material. The Young’s modulus and SSA of the electrodes before and after treatment are compared as measures of mechanical and electrochemical performance, respectively. However, crucial values, such as the change in specific electrode capacitance, are often measured. This fact highlights the necessity to provide this measurement value as well.

While the electrochemical performance of an SSC does not depend exclusively on the electrode porosity, the SSA has a strong correlation with specific capacitance as shown by Shirshova et al. [93]. In Figure 5, most of the presented modifications methods were applied to CF-based electrodes, while some were carried out for other carbon-based materials such as CNT fibers. This explains the comparatively higher SSA of modified CNT fibers. Figure 5 focuses more on demonstrating the improvement in the SSA rather than reporting the absolute values of post-treatment SSA.

It can be seen in Figure 5 that the highest SSA improvement (892 times) for carbon-based fibers is related to the CNT fibers that used sulfonated tetrafluoroethylene based fluoropolymer-copolymer as a pore-forming agent during fabrication (blue data points). An example of combined approaches for SSC electrode improvement is the combination of vertically aligned graphene and manganese dioxide [94] as well as the mixture of CAGs and the CPs using PEDOT [93]. Both methods resulted in a significant increase in specific capacitance, which can be attributed to the synergistic effects of both methods. Moreover, the deposition of either graphene or CAGs not only leads to more porosity, higher SSA, and hence higher specific double-layer capacitance, but also it improves the distribution of manganese dioxide and the CP, which then again contributes to the electrochemical performance by an increase in conductivity and pseudocapacitance. Hudak et al. used a combined approach in which they activated CFs and then deposited both CNTs and PANI. This method led to a 30-fold increase in the specific capacitance and an increase in the flexural strength of 27% with only a 7% decrease in the Young’s modulus [78].

Furthermore, a promising approach to increase the SSA has been the surface deposition of active materials (green markers), such as CAG (384 times) and Cu-Co-Se nanowires (316 times). The CAG surface deposition also resulted in the greatest increase in stiffness (3.2 times), followed by the deposition of GNPs, reaching values between 1.66 and 4.77 GPa (2.9 times), as summarized in Table A1.

There are other potentially promising combined approaches that have not been extensively studied for the use of CF electrodes in SSCs. These approaches include electrochemical and physical activation, heteroatom doping, and the introduction of pores during fabrication, in combination with the widely studied methods of surface deposition of active materials.

### 3.2. Current Collector

Theoretically, for some CF-based electrodes, a current collector might not be necessary. However, especially for long-fiber electrodes, the conductivity is very limited. In these cases, the deposition of ultra-thin metal coatings on the surface of the CF electrodes can help to increase the conductivity of the electrodes [42]. Using copper tape as a current collector, Javaid showed better electrochemical performance in SSCs [95]. Different materials can be used for each electrode, allowing for an asymmetric configuration of the SC. This can improve the working voltage and thus the power and energy densities of the SC [96,97]. However, the introduction of an additional copper tape results in additional weight and thus is contrary to the lightweight approach of SSCs (Figure 2b).

### 3.3. Electrolyte

The electrolyte of an SC acts as the distributor of charges between the two electrodes and therefore plays an important role [98]. The functionality of electrolytes requires a resistance against high voltages with a value of more than 3 V, high ionic conductivity, and a high chemical stability. All these properties influence the SC’s energy and power density. For SCs, a wide range of electrolytes have been extensively investigated including aqueous, organic, redox-type, solid-state, and semi-solid electrolytes and ionic liquids [99]. Aqueous electrolytes, such as KOH, ${\mathrm{Na}}_{2}{\mathrm{SO}}_{4}$, $H_{2}{\mathrm{SO}}_{4}$ and ${\mathrm{NH}}_{4}\mathrm{Cl}$ solutions, offer higher ionic conductivity with values of approximately 1 S ${\mathrm{cm}}^{-1}$ and require a minimum pore size on the electrode surface. However, they are limited by small potentials, reaching values of approximately 1.2 V [100,101]. In contrast, organic electrolytes exhibit lower electrical conductivity, ranging from 10 to 60 mS ${\mathrm{cm}}^{-1}$ but compensate with a wider potential window from 2.5 to 2.7 V [77]. Ionic liquids possess exceptional properties including high thermal and chemical stability, low vapor pressure, a wide potential window, low flammability, and a conductivity of approximately 10 mS ${\mathrm{cm}}^{-1}$ [102].

For usage in structural supercapacitors, electrolytes also need to be able to carry and transfer mechanical loads to the CF electrodes and structural reinforcement layers which requires the use of solid-state electrolytes (SSE) [6]. The three main groups of SSEs include solid polymer electrolytes (SPE), inorganic solid electrolytes (ISE), and hybrid electrolytes (HE) [100]. SPEs are formed by combining a polymer matrix with a lithium salt, resulting in an ion-conducting material, while SPEs such as polyethylene oxide (PEO) exhibit good mechanical properties and show promising applicability for use as a matrix material. However, they have a high ion conductivity, low thermal stability, and low thermal conductivity at room temperature [103]. SPEs have the disadvantage of a high ionic conductivity at room temperature when the electrolyte is in a crystalline condition. At higher temperatures, the electrolyte switches to an amorphous state, which results in a higher ion conductivity due to the increasing dynamics of the amorphous chain and the less tortuous pathways for ion diffusion [104]. However, with an increase in the ratio of fillers to the host material, mechanical properties decrease [95]. ISEs are ceramics that conduct single ions through vacancies and interspaces [103]. Compared to SPEs, their electrochemical performance is thermally stable. The greatest disadvantage of ISEs is a high interface resistivity because of a low charge conductivity due to the lack of flexibility at the electrolyte/electrode interface. Additionally, the high porosity of ISEs make large-scale production difficult [100]. In order to counteract to the contrary features of SPEs and ISEs, HEs are developed by combining both groups of SSEs using (1) inorganic fillers in the polymer matrix, (2) layered SSEs consisting of inorganic and polymeric electrolytes, (3) organic-framework-reinforced electrolytes, or (4) bicontinuous ionic-liquid electrolytes [105].

#### 3.3.1. Solid-State Electrolytes with Inorganic Fillers

Non-conducting passive inorganic fillers, such as ${\mathrm{SiO}}_{2}$, ${\mathrm{Al}}_{2}O_{3}$, and ${\mathrm{TiO}}_{2}$ can be used to reduce the crystallization of the polymer matrix at room temperature. However, these passive nanofillers show low ionic conductivity with values below $10^{-4}$ S/cm. They exhibit adjustable characteristics and are inexpensive and comparatively simple to prepare. In addition, they increase the ionic conductivity of the host material by providing extra ion penetration pathways [106].

In contrast, conducting active inorganic fillers, such as lithium containing fillers, allow ionic flow, not only within the polymer chains but also along the filler’s pathways [106]. Therefore, active fillers have exceptional ionic conductivity reaching values higher than $10^{-3}$ S/cm, but they are difficult to synthesize [107]. Furthermore, rod-shaped active three-dimensional inorganic continuous fillers create fast ion-conducting pathways without crossing junctions along the vertically aligned interfaces of the three-dimensional frameworks, which enables higher ion conductivity [108]. Additionally, vertically aligned and continuous fillers prevent crystallization of the amorphous phases more effectively than regular inorganic fillers [109].

Another promising approach for SSEs is the introduction of self-healing properties by adding inorganic ions, such as KCl and $H_{2}{\mathrm{SO}}_{4}$, which can exhibit self-healing properties. This behavior could counteract the mechanical degradation under mechanical loading and thus could represent an advantageous for SSEs. Thereby, self-healing is achieved due to the formation of hydrogen bonds [110]. To amplify the self-healing properties of the polymer, electrolytes must have high toughness and strength values to support huge deformation due to non-covalent interactions, such as hydrogen bonding [111], coordination [112], dynamic borate ester bonding [113], and host–guest interactions [114]. Testing on a flexible and self-healing supercapacitor based on activated carbon cloth showed outstanding flexibility, a composite capacitance retention of 88% over 10,000 cycles, and a good self-healing capability, with 80% capacitance retention after five cutting/healing cycles [115].

#### 3.3.2. Layered Solid-State Electrolytes

A combination of layers of SPEs and ISEs allows for the adjustment of properties depending on the desired objectives and takes advantage of both electrolyte types to enhance the electrochemical properties of the composite as a whole [116]. This approach is of special interest, when trying to incorporate typical electrolyte systems from battery and supercapacitor research into multifunctional applications. Therefore, layered SSEs can be designed as double layers, with an ion-conducting polymer to reduce interfacial resistance with the anode [117], as a symmetrical sandwiched structure to improve the contact area between the ISE and the electrodes [118] or as an asymmetrical sandwich structure to fulfill multiple demands such as preventing lithium dendrite penetration while simultaneously providing good contact between the electrolyte and the electrodes [119,120]. Huo et al. [121] created a stackup of a polymer–ceramic composite, which achieved high ionic conductivity with values of 0.023 $\mathrm{mS}\cdot {\mathrm{cm}}^{-1}$, while having excellent flexibility and a good tensile strength of 11.3 MPa.

#### 3.3.3. Solid-State Electrolytes with Metal–Organic Frameworks

Improving the electrochemical and mechanical properties of the polymer matrix with organic frameworks can be achieved by employing metal–organic frameworks (MOFs), covalent organic frameworks, and porous organic cages [103]. The enhancement is based on two main principles. The first principle is the containment of crystallization. Due to the Lewis-acidic sites on the MOFs interaction with the polymer chains in the polymer matrix, crystallization can be prevented to a large extent [122]. For the second principle, the incorporation of a highly conductive liquid electrolyte onto the porous surface of the organic frameworks forms a quasi-solid electrolyte, which creates further ion-conducting pathways along the framework [106]. A study by Wang et al. [123] showed a five-fold increase in ionic conductivity and better contact with the electrode than that of the SSE without MOF-related materials. However, mechanical tests on MOF electrolytes are currently rare, making it difficult to quantify their benefit to the overall SSC performance.

#### 3.3.4. Solid State Electrolytes with Bicontinuous Phase Structure

**Figure 6:**
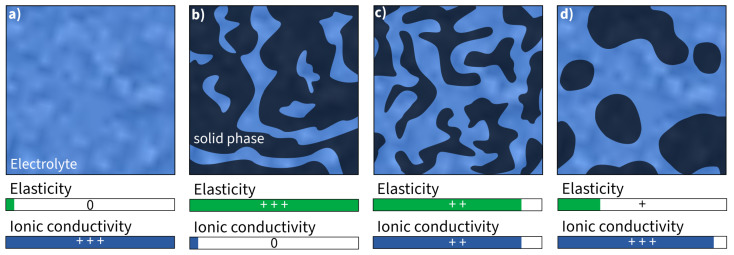
Schematic illustration and assessment of Young’s modulus and ionic conductivity of (**a**) pure liquid electrolyte and (**b**–**d**) different kinds of bicontinuous phase structures (assessment of properties: 0—none, +—good, ++—high, +++—very high).

Bicontinuous phase structures are a way to create polymers that can conduct ions and bear loads at the same time. This is achieved by combining two materials with separate load-bearing and ion conductive functionality [124], which is often performed by synthesizing the polymer electrolyte. This yields a Young’s modulus of approximately 2.77 GPa, a tensile strength of 80 MPa [125], and an ionic conductivity of $10\;\mathrm{mS}\cdot {\mathrm{cm}}^{-1}$ in the presence of an ionic liquid [102]. The presence of an ionic liquid is necessary to form a cross-linking structure in which the ionic liquid is deeply incorporated [126]. Ionic liquids alone cannot transmit any mechanical loads and therefore they reduce the mechanical properties [127,128] (Figure 6). As a result, it is inherently difficult to strike a balance where the improvement of one property is at the expense of the other.

#### 3.3.5. Summary of Different Types of Solid-State Electrolytes

**Figure 7:**
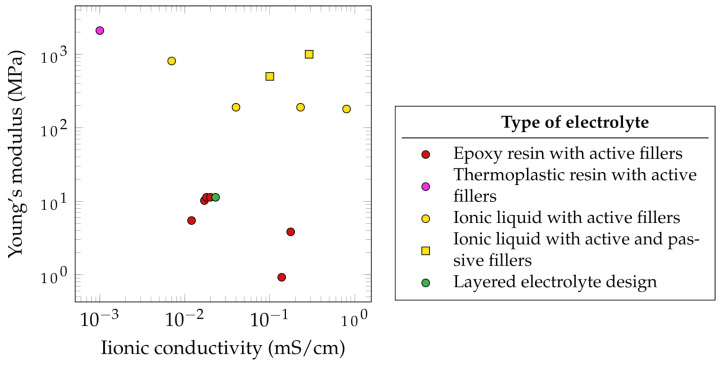
Properties of selected types of electrolyte groups based on Table A2 [95,121,124,131,132].

Figure 7 summarizes the electrochemical and mechanical properties of different SSEs which have been used in SSCs. It has to be noted that the presented parameters, such as the ionic conductivity, depended on several factors. The data from literature do not follow unified standards. Therefore, some standards have been proposed in the literature for evaluating these properties [129]. According to Wang et al., SSEs with ionic conductivities below 1 $\mathrm{mS}\cdot {\mathrm{cm}}^{-1}$ are not applicable for SSCs [130]. Based on this assessment, none of the observed SSEs that have been reviewed fulfill this requirement. Of the reported SSEs, the highest ionic conductivity is 0.8 $\mathrm{mS}\cdot {\mathrm{cm}}^{-1}$, measured for an ionic liquid with active fillers, created by Shirshova et al. [124].

Furthermore, the mechanical properties of SSEs are often significantly lower compared to those of pure epoxy resin. This results in a Young’s modulus of 3.35 GPa and a tensile strength of 86.04 MPa for a CNT/epoxy nanocomposite [133].

Javaid showed that the use of different active fillers such as TBAPF6, LiTFSI, ${\mathrm{NaClO}}_{4}$ and EMITFSI would lead to different ionic conductivities. A similar observation was made by Wang et al., who dissolved a lithium salt in the thermoplastic polymer polyvinylidene fluoride and then mixed it with epoxy resin. A higher epoxy content leads to lower ionic conductivity but higher mechanical properties. Interestingly, when the thermoplastic polymer was mixed with the active filler LiTf and no epoxy was added, the best mechanical and electrochemical properties were observed, suggesting the potential use of thermoplastic electrolytes for SSEs [134]. This approach was further studied by Joyal et al., who created a single-phase solid polymer electrolyte by mixing the thermoplastic polymer polyethylene terephthalate with the lithium salt ${\mathrm{LiClO}}_{4}$ [131], achieving the highest reported Young’s modulus of 2100 MPa but also the lowest ionic conductivity of 1 $\mathrm{mS}\cdot {\mathrm{cm}}^{-1}$. However, the use of thermoplastic polymers as a base for SSEs does not only suggest potential in terms of mechanical and electrochemical properties but also in terms of processability. This could open new ways of fabrication routes for SSCs. Another promising method with high mechanical properties are the use of ionic liquid with active and passive fillers. As an example, DGEBA/LiTFSI/BMIM-TFSI/Al_2_O_4_ (5 vol%) was reported to achieve 1000 GPa and approximately 0.29 $\mathrm{mS}\cdot {\mathrm{cm}}^{-1}$.

The relatively good properties of electrolytes with ionic liquid and active fillers make this approach the most common method of SSE synthesis for SSC application [124,135]. This method allows the active filler to completely dissolve in an ionic liquid such as 1-ethyl-3-methylimidazolium bis(trifluoromethylsulfonyl)imide (EMIM-TFSI), which is then mixed with the epoxy resin. This approach leads to the creation of porous microstructures providing ion path channels to further increase ion conductivity. To additionally increase the mechanical properties of ionic liquid-based SSEs without sacrificing ionic conductivity, Kwon et al. incorporated ${\mathrm{Al}}_{2}O_{3}$ nanowires as passive fillers. Due to the Lewis-acidic conditions of those incorporated ${\mathrm{Al}}_{2}O_{3}$ nanowires, not only the Young’s modulus but also the ion conductivity was enhanced [132].

### 3.4. Separator

**Figure 8:**
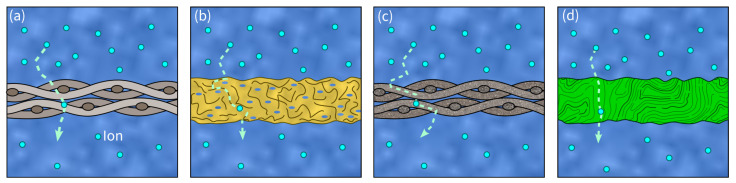
Visualization of different separator materials and ion transport paths (dashed arrows): (**a**) glass fiber separator, (**b**) polymer separator, (**c**) ceramic separator, (**d**) cellulose separator.

An SC’s separator is placed between the two electrodes and prevents electron flow, blocking a short circuit. Therefore, it must provide both high ionic conductivity and mechanical stability [136]. Consequently, in load- carrying SSEs, a separator is essentially not required. However, due to safety concerns, they might still be included in the design. Further important properties in separators are their chemical and thermal stability, low density, corrosion resistance as well as their low cost and availability [6]. Additionally, optimal porosity and minimal thickness for good electrolyte storage and high electrolyte uptake are often linked to high ionic conductivity [6]. The most widely used separator material in SSC is glass fiber. Other promising separators used in conventional SCs are polymers, ceramics, and cellulose [137] (see Figure 8).

#### 3.4.1. Glass Fiber-Based Separators

Woven glass fabrics are the most common material for separators in SSCs [17] due to their excellent mechanical properties [138]. However, they present a higher thickness compared to polypropylene (PP) separators [17], which results in a relatively high area density and therefore a lower specific capacitance [139].

#### 3.4.2. Polymer-Based Separators

Polymer-based separators produced via electrospinning followed by phase separation and phase inversion processes show very good electrochemical performance compared to commercial, glass fiber-based separators. Due to their high porosity and SSA, the electrolyte is not only adsorbed on the surface but also penetrates the porous structure, leading to a much higher electrolyte uptake, which increases the ionic conductivity. While separators with a higher porosity lead to improved electrochemical performance, their porosity weakens the mechanical properties if used in an SSC [140]. Karabelli et al. used the phase inversion method to create porous separators with good mechanical properties. While dense polymer membranes have shown a Young’s modulus of 741 MPa, the same material with 75% porosity only exhibits a Young’s modulus of 53 MPa [141]. Consequently, the mechanical properties of polymer-based separators stand far behind the mechanical properties of glass fiber-based separators and thus are not recommended for use in SSCs.

#### 3.4.3. Gel and Solid Polymer-Based Electrolyte Separators

Gel and solid polymer-based electrolyte separators offer great potential for use in SSCs since they can function as the electrolyte and the separator at the same time. Consequently, an extra separator is not needed, resulting in weight reduction, simpler design, and easier fabrication route in the energy storage devices [140]. Ma et al. used the gel polymer $\mathrm{PVA}-\mathrm{KOH}-K_{3}\left[\mathrm{Fe}{\left(\mathrm{CN}\right)}_{6}\right]$ functioning as an electrolyte and separator, creating a flexible and stretchable SSC [142]. While gel polymer-based electrolyte separators are eligible for application in flexible SSCs, the mechanical properties of such devices cannot compete with those of CFRP composites. Therefore, solid polymer-based electrolytes acting as separators offer greater potential for stiff SSCs. Hubert et al. developed an SSC using solid electrolytes. Additionally, they assembled another SSC with the same electrolyte but this time added a cellulose-based separator (TF40-30, NKK Nippon Kodoshi, Koshi, Japan). Though the results of electrochemical tests proved the concept of solid polymer-based electrolyte separators, they also showed that the specific capacity of the mentioned device at 623 mF/g is much lower compared to the same SSCs with a traditional separator at 1.44 F/g. On the other hand, mechanical tests of the separator-free SSC propose good mechanical properties comparable to other SSCs [139].

#### 3.4.4. Polymer–Ceramic-Based Separators

A different method of employing polymers as separators in energy storage devices is the use of polymer-ceramic-based separators [56,140]. Adding ceramic nanoparticles to a polymer can drastically improve its ion conductivity and enhance its thermal stability [143]. The improvement in ion conductivity is achieved thanks to the creation of amorphous regions in the polymer matrix, which leads to a more porous structure [144]. In addition to improving the electrochemical properties of SCs, the use of ceramic nanoparticles in polymer electrolyte separators can also improve mechanical properties. By using polymer/ceramic composites based on polyvinylidene fluoride (PVDF) and PP mixed with different alkaline earth metal-based titanites, Alvarez-Sanchez et al. showed that the mechanical and electrochemical performance of polymer–ceramic-based separators are improved compared to those of traditional separators [145]. The concept of polymer–ceramic-based separator has already been proven for SSE in lithium–sulfur batteries and therefore it is expected to be relevant for SSE in SSCs [146].

#### 3.4.5. Ceramic Separators

Due to their outstanding thermal stability, ceramic separators have been used in different applications for high-temperature SCs and thermal batteries [147,148]. At high operational temperatures, regular liquid electrolytes used in SCs are not suitable because of their relatively low boiling temperatures [149]. For this reason, ceramic separators are used with solid electrolytes and ionic liquids [140], which are one of the most common electrolyte composition used in SSCs [45,150]. Ceramic separators for SCs provide excellent thermal, physical, and electrochemical properties. While mechanical properties for separators in regular SCs are important, they do not need to be extraordinarily high. In SSCs, however, the current state-of-the-art ceramic separators show inadequate tensile strength [147].

Another approach in which ceramic-based separators can be used is the development of ceramic fibers working as the separator and as reinforcement of the electrolyte matrix. Zhao et al. created a working ceramic fiber separator for lithium-ion batteries by incorporating short-section irregular ceramic fibers into the matrix [151], consolidating matrix materials with continuous ceramic fibers and improving the composite strength. Owing to the exceptional mechanical properties of ceramic fibers, an increase in strength and Young’s modulus compared to other SSCs using glass fiber separators can be expected. This is due to the fact that SiC-based fibers present tensile strengths of up to 6 GPa and Young’s modulus values of up to 420 GPa [152]. In addition, Yamamoto et al. developed a method to grow aligned CNTs on ceramic fibers, which can further enhance the bonding between fiber and matrix and demonstrate the potential to enlarge the porosity of the ceramic fiber separator to increase ionic conductivity [153].

#### 3.4.6. Cellulose-Based Separators

Plenty of studies have analyzed the use of cellulose-based separators for SCs and have shown promising results for high-performance EESs [154,155]. Additionally, Xu et al. showed that the mechanical properties of SCs with cellulose-based separators would increase at a higher cellulose concentration. Tensile strengths of 76.02 MPa and a Young’s modulus of almost 6 GPa were observed. While these properties are relatively low compared to GF separators, they suggest a potential use for cellulose-based separators in flexible SSCs [156]. In this context, successful tests with cellulose-based hydrogels as recyclable electrolytes have been performed and have achieved a long life cycle and 92% capacitance retention after 10,000 consecutive voltammetry cycles, which was higher than similar designs using the reference $\mathrm{PVA}/H_{3}{\mathrm{PO}}_{4}$ gel electrolyte [157]. Further potential for SSCs is given by the use of mesoporous nanocellulose membrane separators [158] owing to the outstanding mechanical properties of nanocellulose, which can reach a Young’s modulus of up to 130 GPa [159].

#### 3.4.7. Summary of Different Approaches for Separators Used in SSCs

**Figure 9:**
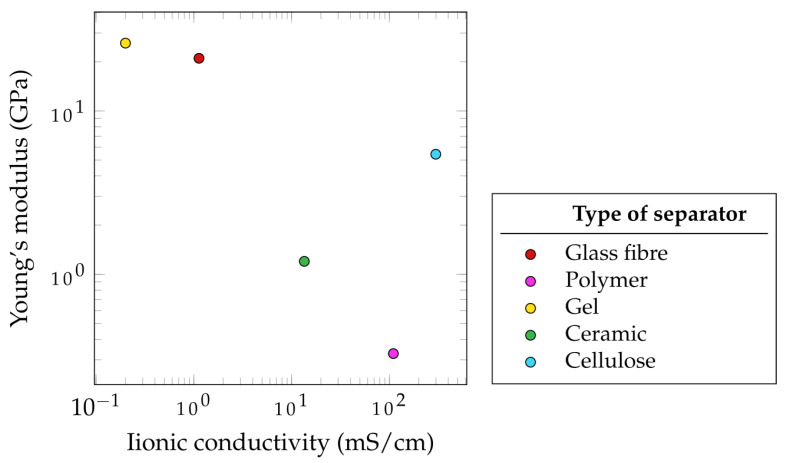
Properties of selected types of separators based on Table A3 [135,139,140,145,156,160,161,162].

A common separator for SSC applications is SSC application is glass fiber, with a Young’s modulus of 21 GPa, but also one of the lowest ionic conductivity values, with 1.13 $\mathrm{mS}\cdot {\mathrm{cm}}^{-1}$ [135]. The high mechanical properties are only surpassed by solid polymers, achieving 26 GPa, which show six times less ionic conductivity [139,160] (Figure 9). A higher ionic conductivity is achieved with ceramic-based separators (13.5 $\mathrm{mS}\cdot {\mathrm{cm}}^{-1}$) [161]. This property could be further improved with the use of a polymer-based RF/PLA combination resulting in nine times higher measurements [162]. However, the highest value was achieved with cellulose (298.6 $\mathrm{mS}\cdot {\mathrm{cm}}^{-1}$). Additionally, a bio-inspired separator also showed good mechanical properties (5.43 GPa) [140,156].

Even though the mechanical property values of GF are up to four times higher than cellulose-based separators, the research into these bio-based designs could be of great importance, especially with new challenges arising, such as recyclability and environmental friendliness of new SSC developments.

## 4. Multifunctionality Analysis in Current SSCs and Future Work

**Figure 10:**
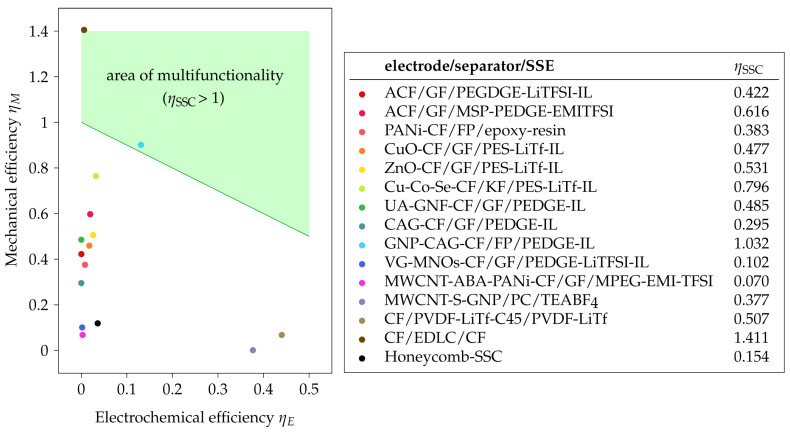
Multifunctionality of selected SSCs based on Table A4 [11,21,45,76,78,84,86,87,94,134,135,163,165,166,167].

Considering the multifunctional performance of a selected group of SSCs, it can be observed that only two of the reported SSCs (Figure 10, Table A4) achieve a multifunctional efficiency value above 1 according to the benchmark values earlier (see Section 2.2). The first SSC is comprised of CNT fiber veils and an ionic liquid-based polymer electrolyte embedded in CF plies (cf. [8]), achieving excellent mechanical properties but negligible electrochemical performance. The second promising SSC uses a combination of CAGs and GNPs to coat the CF electrodes (cf. [87]) and obtained a more balanced performance in both domains. Furthermore, there are two SSCs with the electrode/separator/SSE configuration MWCNT-S-GNP/PC/TEABF4 (cf. [163]) and CF/PVDF-LiTf-C45/PVDF-LiTf (cf. [134]), which had significantly higher electrical properties (Figure 10) but still had a low electrochemical efficiency (below 0.5) in comparison to conventional SCs. In contrast, the remaining SSCs exhibit much higher mechanical efficiency than electrochemical efficiency.

The observation and assessment of the multifunctionality of different reported SSCs shows that while a lot of progress has been already made, there are still many open approaches to further advance the multifunctional performance of SSC. One approach which is seemingly overlooked consists of SSCs with a higher electrochemical performance. A promising example might be a combination of CAGs and a GNP-doped CF electrode together with an epoxy-based adhesive polymer electrolyte, which has reportedly gained multifunctionality with high electrochemical efficiency.

However, further combinations of materials and modifications are under investigation. High-performing SSCs in terms of mechanical and overall efficiency achieve this by improving structural electrolyte properties and using activated CFs. Further, the deposition of active materials, i.e., deposition of nickel oxide [164], on the activated CF-based electrodes of the SSC can provide remarkable overall efficiency. This significantly increases the pseudocapacitance. However, the used approach is only one of many possible steps to further improve the electrochemical efficiency of CF electrodes [83]. For the electrolyte systems, the use of ionic liquid with active and passive fillers (cf. [132]) provides very good ionic conductivity and also an acceptable Young’s modulus. Changing the ratio of liquid and solid phase in the bicontinuous electrolyte can be used to further adjust the mechanical and electrochemical properties. In addition, replacing the glass fiber separator with cellulose (cf. [140,156]) could also result in better conductivity.

However, an accurate assessment of SSC requires a unified reporting strategy of the required data and standardization in experimental testing. Mechanical tests, such as tensile and flexural tests, are especially required. Furthermore, experimental analyses of the coupled electrochemical–mechanical phenomena should be the scope of future investigations.

## 5. Conclusions

Structural supercapacitors (SSCs) play an important role in high-power-demand lightweight applications. Each use case has different SSC requirements in terms of mechanical and electrical performance, but the overall weight reduction is only dependent on the combined multifunctional efficiency. The observation and assessment of the multifunctionality of different reported SSCs shows that while more improvement is still required, there are also many promising approaches to further advance the multifunctional performance of SSCs.

From the reported SSCs, two reported approaches achieved a relevant multifunctional efficiency based on the selected benchmark values. The first uses CNT fiber veils and an ionic liquid-based polymer electrolyte embedded in CF plies, resulting in excellent mechanical properties but negligible electrochemical performance. The second SSC uses a combination of CAGs and GNPs to coat the CF electrodes and obtained a more balanced performance in both domains. Furthermore, this review showed that in general more SSCs have higher mechanical efficiency and lower electrochemical properties than conventionally available SSCs. The adding of additional CF plies to increase the mechanical properties is a simple approach to improve the mechanical efficiency of SSCs.

To achieve SSCs with higher electrochemical performance, the optimization and combination of favorable materials is an important step. In this review, multiple starting points for this endeavor have been identified. For the electrodes, the deposition of active materials on activated carbon fiber electrodes has showcased a high overall efficiency. Furthermore, the use of ionic liquid with active and passive fillers lead to very good ionic conductivity and also an acceptable Young’s modulus. Moreover, by changing the ratio of liquid and solid phase in the bicontinuous electrolyte, the mechanical and electrochemical properties can be adjusted to specific use cases. In addition, replacing the glass fiber separator with cellulose resulted in better conductivity and could be an important step into improving the recyclability of SSCs.

In order to fully exploit the potential of newly developed systems, it is crucial to have access to comprehensive and standardized measurements of the electrochemical and mechanical properties. Nevertheless, a lack of sufficient characterization and analysis of these materials complicates the selection of suitable constituents and impedes the realization of their full potential. Therefore, it is imperative for future research endeavors to characterize the SSCs with standardized tests, including: (1) classical mechanical testing, such as tensile tests in the longitudinal (fiber) and transversal directions and four-point bending tests, (2) conventional electrochemical tests, such as cyclic charge and discharge and impedance spectroscopy, (3) standardized coupled testing which would provide combinations of static and dynamic mechanical and electrical loads to see the in situ response of the SSC under various load cases including cycling/fatigue behavior. These tests would allow for a better understanding and the prediction of SSC performance in real life applications.

## Figures and Tables

**Table 1:** Comparison between different carbon fiber fabrics and their properties.

	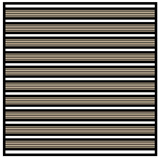	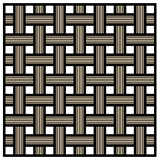	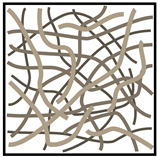
CF	Unidirectional	Woven	Non-Woven
Load bearing capability *	++	++	+
Specific surface area *	+	+	++
Electric conductivity *	+	+	0

* Assessment of properties: 0 — none, + — good, ++ — high.

## Data Availability

The data presented in this study are available on request from the corresponding author.

## Abbreviations

The following abbreviations are used in this manuscript:

EDLC	Electric double-layer capacitor
EES	Electrical energy storage
ESD	Energy storage devices
CAG	Carbon aerogel
CF	Carbon fiber
CFRP	Carbon fiber reinforced polymers
CNT	Carbon nanotube
CP	Conducting polymer
FRC	Fiber reinforced composites
GF	Glass fibers
GNP	Graphene nanoplatelets
GO	graphene oxide
HE	Hybrid electrolyte
ISE	Inorganic solid electrolyte
LiB	Lithium-ion battery
MESC	Multifunctional energy storage composite
MOF	Metal–organic framework
Ni	Nickel
SC	Supercapacitor
SPE	Solid polymer electrolyte
SSA	Specific surface area
SSC	Structural supercapacitor
SSE	Solid-state electrolyte
UD	Unidirectional

## Appendix A. Data Tables

**Table A1:** Properties of different modified CF-based electrodes.

Type of Modification		Untreated SSA * (m^2^/g)	Treated SSA * (m^2^/g)	Redox Activity	Influence on Stiffness of the CF *	Influence on Interfacial Shear Strength *	Ref.
Thermo-chemical activation	CF/KOH	0.331	32.8	No	Slight overall increase	n.a.	[168]
Wet chemical activation	CF/KMnO_4_/H_2_SO_4_	6	92	No	Slight overall decrease	Increase	[54]
Electrochemical activation	N/O-enriched carbon cloth	419.85	468.59	Yes	Decrease in tensile strength	Increase	[57]
Physical activation	Plasma-treated active carbon fibers	1500	2103	Yes	Overall increase [88]	n.a.	[61]
Heteroatom-doping	Boron and oxygen co-doped carbon nanofibers	461.5	725.7	Yes	Increase in tensile strength with a decrease in young’s modulus [90]	n.a.	[89]
Introduction of pores during fabrication	Carbonization and activation of carbon nanofibers in CO_2_	294	705	Yes	Overall decrease	Increase	[49]
	Sulfonated tetrafluoroethylene based fluoropolymer-copolymer as pore-forming agent for carbon nanofibers	339	1614	No	Overall decrease	Increase	[50]
Surface deposition of active materials	CF/CNT sizing	0.21	33.4	No	Decrease in tensile strength	Increase [74]	[91]
	CF/CNT grafting	0.21	45.8	No	No significant effects on modulus [74]		
	CF/GNP	0.33	n.a.	No	No significant effects	Increase	[76]
	CF/conducting polymers	n.a.	n.a.	Yes	No significant effects	No significant effects	[77]
	CF/Cu-Co-Se nanowires	0.62	195.65	Yes	n.a.	Increase	[84]
	CF/CAG	0.21	80.7	No	n.a.	Increase	[86]
Combination	CF/CAG/GNP	0.25	223	No	n.a.	Increase	[87]

* The abbreviation not available (n.a.) is used.

**Table A2:** Properties of selected types of solid-state electrolytes.

Type of Electrolyte	Composition	Ionic Conductivity * (mS/cm)	Young’s Modulus * (MPa)	Tensile Strength * (MPa)	Ref.
Epoxy resin with active fillers	PEGDGE/TBAPF_6_/PC	0.012	5.46	n.a.	[95]
	PEGDGE/LiTFSI/PC	0.017	10.2	n.a.	
	PEGDGE/NaClO_4_/PC	0.018	11.3	n.a.	
	PEGDGE/EMITFSI	0.020	11.3	n.a.	
	DGEBA/LiTFSI/PC	0.138	0.922	n.a.	
	PEGDGE/EMITFSI	0.176	3.83	n.a.	
	Epoxy Resin/[PVDF/LiTf (25:75)]	10.8	n.a.	80	[134]
	Epoxy Resin/[PVDF/LiTf (25:75)]	24.8	n.a.	55	
Thermoplastic resin with active filler	PVDF/LiTf (25:75)	39.6	n.a.	66	[134]
	28.9	n.a.	136		
	PET/LiClO_4_ (90:10)	0.001	2100	n.a.	[131]
Ionic liquid with active fillers	MVR444/EMIM-TFSI/LiTFSI	0.04	190	n.a.	[124]
	MVR444/EMIM-TFSI/LiTFSI	0.23	190	n.a.	
	MTM57/EMIM-TFSI/LiTFSI	0.007	810	n.a.	
	MTM57/EMIM-TFSI/LiTFSI	0.8	180	n.a.	
Ionic liquid with active and passive fillers	DGEBA/LiTFSI/BMIM-TFSI	~0.1	500	n.a.	[132]
	DGEBA/LiTFSI/BMIM-TFSI/Al_2_O_4_ (5 vol%)	~0.29	1000	n.a.	
1Layered electrolyte designs	hierarchical ${\mathrm{Li}}_{6.4}{\mathrm{La}}_{3}{\mathrm{Zr}}_{1.4}{\mathrm{Ta}}_{0.6}O_{12}$ sandwich	0.023	11.3	n.a.	[121]

* The abbreviation not available (n.a.) is used.

**Table A3:** Properties of different types of separators.

Type of Separator	Separator Material	Ionic Conductivity * (mS/cm)	Young’s Modulus * (GPa)	Strength * (MPa)	Ref.
Glass fibre	Glass fibre	1.13	21	325	[135]
Polymer	RF/PLA	110	0.3271	15.2	[161]
Solid polymer electrolyte	$\mathrm{PEGDGE}/\mathrm{TETA}/{\mathrm{EMIBF}}_{4}$	0.2	26	350	[139,160]
Ceramic	$\mathrm{PVDF}/\mathrm{PPG}/\mathrm{LiCl}/{\mathrm{CaTiO}}_{3}$	n.a.	1.2	65	[145]
	$\mathrm{PVB}/{\mathrm{Al}}_{2}O_{3}\mathrm{NW}$	13.5	n.a.	30	[162]
Cellulose	MCC/AMIM-Cl	298.6	5.43	71.71	[140,156]

* The abbreviation not available (n.a.) is used.

**Table A4:** Mechanical and electrochemical properties of some selected SSCs.

SSC Assembly	Specific Capacitance * (F/g)	Power Density * (W/kg)	Energy Density (mWh/kg)	Modulus ^1^ (GPa)	Strength ^2,^* (MPa)	Ref.
ACF/GF/PEGDGE-LiTFSI-IL	0.052	2.68	1.43	$E_{\left|\right|}$ = 18.04	n.a.	[45]
ACF/GF/MSP-PEDGE-EMITFSI	n.a.	34.4	117.7	$G_{||⊥}$ = 1.75	$R_{||⊥}$ = 38.2	[76]
PANi-CF/FP/epoxy-resin	0.022	58.4	49.4	$G_{||⊥}$ = 1.1	$R_{||⊥}$ = 6.3	[165]
CuO-CF/GF/PES-LiTf-IL	6.75	12.57	106.04	$E_{\left|\right|}$ = 19.62	$R_{\left|\right|}^{t}$ = 251.76	[166]
ZnO-CF/GF/PES-LiTf-IL	18.82	19.87	156.21	$E_{\left|\right|}$ = 21.59	$R_{\left|\right|}^{t}$ = 325.82	[135]
Cu-Co-Se-CF/KF/PES-LiTf-IL	28.63	36.65	191.64	$E_{\left|\right|}$ = 32.65	$R_{\left|\right|}^{t}$ = 488.89	[84]
UA-GNF-CF/GF/PEDGE-IL	0.048	0.788	0.067	$E_{\left|\right|}$ = 20.72	$R_{\left|\right|}^{t}$ = ~90	[167]
CAG-CF/GF/PEDGE/IL	0.071	0.033	0.099	$G_{||⊥}$ = 0.895	$R_{||⊥}$ = 8.71	[86]
GNP-CAG-CF/FP/DGEBA-${\mathrm{LiClO}}_{4}$	0.354	107.8	786.05	$G_{||⊥}$ = 2.64	$R_{||⊥}$ = 8.70	[87]
VG-MnO_2_-CF/GF/PEGDGE-LiTFSI-IL	240	2.21	12.2	$E_{\left|\right|}$ = 4.3 **	$R_{\left|\right|}^{t}$ = 87 **	[94]
MWCNT-ABA-PANi-CF/GF/MPEG-EMI-TFSI	0.125	n.a.	17.4	$E_{\left|\right|}$ = 2.9 ^3^	$R_{\left|\right|}^{t}$= 21 ^3^	[78]
MWCNT-S-GNP/PC/${\mathrm{TEABF}}_{4}$	12.2	82.2	2260	$E_{\left|\right|}$ = 0.037 ^3^	$R_{\left|\right|}^{t}$= 0.24 ^3^	[163]
CF/PVDF-LiTf-C45/PVDF-LiTf	69.68	n.a.	2640	$E_{\left|\right|}$ = 2.9 **	$R_{\left|\right|}^{t}$ = 80	[134]
CF/EDLC(CNT fiber/thermoplastic-PYR14-TFSI)/CF	0.088	30	37.5	$E_{\left|\right|}$ = 60 ^3^	$R_{\left|\right|}^{t}$ = 153 ^3^	[8]
Honeycomb-SSC	158.7	11.09	216.7	$E_{\left|\right|}$ = 5.07 ^3^	$R_{\left|\right|}^{t}$= 413.9 ^3^	[21]

^1^ Value represents elastic modulus according to the following notation: Young’s modulus of a unidirectional lamina parallel $E_{\left|\right|}$ and shear modulus transverse-parallel $G_{||⊥}$. ^2^ Value represents elastic modulus according to the following notation: interlaminar shear strength $R_{||⊥}$ and $R_{\left|\right|}^{t}$. ^3^ Values obtained from flexural tests. * The abbreviation not available (n.a.) is used. ** Determined by digitalization and own analysis of the presented data.
